# Gastroesophageal reflux and PPI exposure alter gut microbiota in very young infants

**DOI:** 10.3389/fped.2023.1254329

**Published:** 2023-10-31

**Authors:** Denease Francis, Anupama Chawla, Joseph F. LaComb, Katherine Markarian, Charles E. Robertson, Daniel N. Frank, Grace N. Gathungu

**Affiliations:** ^1^ Department of Pediatrics, Texas Tech University Health Sciences Center El Paso, El Paso, TX, United States; ^2^ Department of Pediatrics, Stony Brook University Hospital, Stony Brook, NY, United States; ^3^ Department of Medicine, University of Colorado Anschutz Medical Campus, Aurora, CO, United States

**Keywords:** gastroesophageal reflux, infants, proton pump inhibitors, microbiome, dysbiosis

## Abstract

**Importance:**

Infants with symptomatic Gastroesophageal reflux are treated with pharmacological therapy that includes proton pump inhibitors (PPI) with clinical improvement. The alterations to gut microbiome profiles in comparison to infants without reflux is not known.

**Objective:**

To determine the effect of PPI therapy on gut bacterial richness, diversity, and proportions of specific taxa in infants when compared to infants not exposed to acid suppressive therapy.

**Design, setting, and participants:**

This cohort study was conducted at the Stony Brook Hospital in Stony Brook, NY between February 2016, and June 2019. Infants meeting inclusion criteria were enrolled in a consecutive fashion.

**Results:**

A total of 76 Infants were recruited and 60 were enrolled in the study, Twenty nine infants met clinical criteria for reflux and were treated with PPI therapy: median [IQR] gestation: 38.0 weeks [34.7–39.6 weeks]; median [IQR] birthweight: 2.95 Kg [2.2–3.4]; 14 [46.7%] male) and 29 infant were healthy controls median [IQR] gestation: 39.1 weeks [38–40 weeks]; median [IQR] birthweight: 3.3 Kg [2.2–3.4]; 17 [58.6%] male); 58 stool samples from 58 infants were analyzed. There were differences in Shannon diversity between the reflux and control groups. The reflux group that was exposed to PPI therapy had increased relative abundance of a diverse set of genera belonging to the phylum Firmicutes. On the other hand, the control group microbiota was dominated by *Bifidobacterium*, and a comparatively lower level of enrichment and abundance of microbial taxa was observed in this group of infants.

**Conclusions and relevance:**

We observed significant differences in both α- and β-diversity of the microbiome, when the two groups of infants were compared. The microbiome in the reflux group had more bacterial taxa and the duration of PPIs exposure was clearly associated with the diversity and abundance of gut microbes. These findings suggest that PPI exposure among infants results in early enrichment of the intestinal microbiome.

## Introduction

Gastroesophageal reflux (GER) results in movement of gastric contents into the esophagus with or without vomiting (1) and occurs in most infants. This improves with age after normal lengthening of the esophagus, solid food intake, and reduction in transient lower esophageal sphincter (LES) relaxation (1). In contrast, gastroesophageal reflux disease (GERD) is defined as reflux with bothersome symptoms or complications (1). Treatment includes lifestyle changes, medications, or surgery. The main agents used in infants are histamine-2 receptor antagonists (H-2 antagonists) and proton pump inhibitors (PPIs) (2). PPIs are considered superior to H-2 antagonists for the treatment of GERD. With an increase in use of PPIs among infants, concern has arisen that PPI use may have unknown effects on overall health.

There is increasing awareness of the relationship between the human gut microbiota and our health (3, 4). The processes for development and maturation of gut microbiota are not fully understood. Evidence suggests that nutritional interventions during pregnancy may play a role (5). Other factors are the gestational age at birth (6), mode of delivery (7, 8), feeding routine (breast or formula) (9), maternal health (10, 11), genetics, exposure to antibiotics in early childhood, and environmental exposures (12, 13).

Alterations in gastric pH, such as those induced by use of PPIs, may influence the gut microbiota profile. A case-control study by Gupta et al., examined the effect of H-2 antagonists on the fecal microbiota of premature infants and found that they decreased the gut microbial diversity leading to a predominance of Proteobacteria (4). This is further supported by an adult study performed by Tsuda et al. (14–16). A small but significant change in fecal microbial diversity was noted including a decrease in Faecalibacterium, a bacteria known to have anti-inflammatory effects (14). In another study the impact of PPI use on gut microbiota was described to be more severe than antibiotics and other common drugs (17). Among children, the long-term use of PPIs has been associated with increased risk of obesity (18).

Based on the limited data we hypothesized that infants treated for GERD with PPI would have an altered microbiome when compared to infants not exposed to acid suppressive therapy. We carried out a case-controlled study enrolling seventy-six infants in the age group of 2 weeks to 6 months. The primary goal of the study was the assessment of PPI exposure on gut microbiome diversity in very young infants. Fecal bacteria were identified and enumerated using 16S rRNA gene sequencing.

## Methods

### Patient population

All infants were recruited prospectively through the Stony Brook Primary Care and Pediatric Gastroenterology outpatient center. Parents gave written informed consent, and infants were enrolled in a consecutive fashion. Seventy-six infants were recruited between February 2016 and June 2019. Infants with a clinical diagnosis of reflux were enrolled if they were on PPI therapy for at least 4 weeks prior to enrollment. Eligibility criteria included age between 2 weeks and 6 months. Infants in the control arm had no prior exposure to reflux medications and were otherwise healthy. The exclusion criteria for all infants were congenital malformations including digestive defects or intestinal surgery, and antimicrobial use within 30 days of study enrollment. Demographic data including gestational age, birth weight, age and weight at enrollment, ethnicity, infant diet, mode of delivery, medications, and maternal dietary restrictions was collected.

### Fecal samples

Enrollees were provided a prepaid fecal sample collection kit containing RNAlater, gloves, ice packs, and a specimen bag. For each subject a single fecal specimen in RNAlater was received, within 24 h of collection. Seventeen subjects did not complete the sample collection and were excluded from the study. The stool samples were stored at −80^°^C until batch processing.

### Microbiota profiling

DNA extraction used the Fecal DNA MiniPrep kit (Zymo Research, Inc) as per manufacturer's recommendations. The bacterial 16S ribosomal RNA gene V3-V4 region was PCR amplified using dual-indexed primers: 338F (5′ ACTCCTACGGGAGGCAGCAG) and 806R (5′ GGACTACHVGGGTWTCTAAT). PCR products were normalized using a SequalPrep^TM^ kit (Invitrogen, Carlsbad, CA), pooled, and purified using a DNA Clean and Concentrator Kit (Zymo, Irvine, CA). The pool was quantified using Qubit Fluorometer 2.0 (Invitrogen, Carlsbad, CA), diluted to 4nM, and denatured with 0.2 N NaOH. Illumina paired-end sequencing was performed on the MiSeq using a 600-cycle v3 reagent kit. 16S rRNA gene sequences were quality filtered and classified using the SINA/SILVA platform (19, 20) as previously described (21, 22). 16S rRNA sequences were taxonomically binned based on lowest common ancestor using SINA/SILVA's default parameters. A median of 102,702 (IQR 56,610–134,490) sequences per sample was generated for the set of 58 samples analyzed. All sequence libraries had a Good's coverage index >99%, indicating excellent depth of coverage. Sequence data and clinical/demographic metadata were deposited in the NCBI Sequence Read Archive under BioProject accession number PRJNA887191.

### Statistical analysis

Rare taxa, defined as those observed in <10% of subjects or with maximum percent relative abundance <0.1% across all subjects were excluded from analysis. Differences in overall microbiota composition (β-diversity) between groups were assessed by permutational analysis of variance (PERMANOVA) tests using Morisita-Horn dissimilarity scores with 10^6^ permutations. For principal coordinates analysis (PCoA) dissimilarity matrices were calculated using the Morisita-Horn index (vegdist function of vegan R package) (23) and applied to the cmdscale function in R. Standard measures of αα-diversity, including richness (Sobs), community evenness (Shannon H/Hmax), and diversity (Shannon diversity, H), were estimated at the rarefaction point of 20,000 sequences through 1,000 re-samplings. Results were assessed by analysis of variance tests. Individual taxa differing between groups were identified using the ANOVA-like differential expression (ALDEx2) R package (24, 25). *P*-values were corrected for multiple comparisons using false discovery rate (26). Effect size plots are derived from ALDEx2 and represent the median effect sizes, calculated as the median between-group difference in CLR values between groups divided by the largest within-group difference in CLR values (24, 25). Descriptive statistics were obtained for all study variables, as appropriate for the data type.

## Results

The clinical characteristics of infants treated with PPI medication (*N* = 30) and unaffected controls (*N* = 29) are detailed in Table 1. PPI treated infants are henceforth called Reflux infants. Overall, control subjects were significantly younger (*P* = 0.004), however, mean weight at enrollment was similar: 5.74 Kg vs. 6.39 Kg in control and Reflux subjects, respectively. Reflux infants were mostly Caucasian race. There was an equal gender distribution, 41.4% females compared to 53.3% females among controls and Reflux infants, respectively (*P* = 0.44). Reflux infants had higher proportions of cesarean delivery (*P* = 0.0092). The proportion of breast-fed infants was higher among controls, 35.8% compared to 3.3%, while the proportion of formula fed infants was higher among Reflux infants (86.7% vs. 51.7%, respectively) and this was statistically significant (*P* = 0.0054). Gestational age was significantly lower among Reflux infants (*P* = 0.0056) and this corresponded to significantly lower mean birth weights in the same group (*P* = 0.0083). Most infants with reflux consumed cereal (70%) and only 6.9% of controls. 84% of reflux infants had confirmed prior exposure to H2-antagonist medication.

**Table 1:** The characteristics of cases and controls.

	Controls	Reflux	*p* value
Number of patients N (%)	30 (50.8)	29 (49.2%	
Enrollment age (months)	0.6–6.0	2.0–6.0	0.0004
Mean weight (Kg)	5.74	6.39	0.1011
Ethnicity			0.0009
Caucasian	13 (44.8%)	26 (86.7%)	
Hispanic	6 (20.7%)	0	
Asian	1 (3.4%)	1 (3.3%)	
Black	2 (6.9%)	2 (6.7%)	
Turkish	0	1 (3.3%)	
Filipino	1 (3.4%)	0	
Multi-racial	6 (20.7%)	0	
Gender			0.4379
Female	12 (41.4%)	16 (53.3%)	
Male	17 (58.6%)	14 (46.7%)	
Mode of delivery			0.0092
C-section	10 (34.5%)	21 (70%)	
Vaginal	19 (65.5%)	9 (30%)	
Infant diet			0.0054
Breast fed	10 (35.8%)	1 (3.3%)	
Formula	15 (51.7%)	26 (86.7%)	
Mixed	4 (13.3%)	3 (10%)	
Maternal diet			0.1253
Restricted	9 (31%)	4 (13.3%)	
Not restricted	20 (69%)	26 (86.7%)	
Gestational age (weeks)	31.4–42	26–40	0.0056
Mean birth weight (Kg)	3.28	2.79	0.0083
Cereal N (%)	2 (6.89)	21 (70)	<0.0001
Zantac N (%)	0	25 (83.33)	N/A

Statistical analysis was conducted in GraphPad prism version 5.04 (San Diego, CA). Fisher Exact, Chi-square, independent t-test were used to analyze the data. Ethnicity was analyzed in 2 groups of Caucasians and others due to small numbers in other racial groups.

Fecal microbiotas were profiled in 58 infants (29 with Reflux, 29 controls) and analyzed in terms of microbial diversity (a- and β-diversity) and differential abundances of individual taxa. We first assessed relationships between clinical/demographic variables and overall microbiome composition (β-diversity) by univariable PERMANOVA (Table 1). Significant associations were observed for disease status (*P* = 0.0078), age (*P* = 5.0e^−6), maternal diet restriction (*P* = 0.021), and enrollment weight (*P* = 0.00014). Notably, maternal diet restrictions were varied among the 13 mothers. This encompassed single or multiple foods including dairy, soy, eggs, nuts, broccoli-cauliflower for one mother, and low-carbohydrate diet for 2 mothers. In a multivariable model combining these four variables, only disease status (*P* = 0.0048) and age (*P* = 0.011) remained significant after adjusting for the other variables. Consequently, age was added as a covariate in subsequent analyses. Other variables, including breastfeeding status (*P* = 0.00025), H-2 antagonists use (*P* = 0.010), and cereal consumption (*P* = 0.0025) also were significant in univariable PERMANOVA tests (Table 2). However, as described above, the proportions of infants who were breastfed, exposed to H-2 antagonists, or consumed cereal were heavily skewed between Reflux and Control groups, so these results likely were confounded by disease status. Rather than including these variables as covariates in PERMANOVA analyses, we performed stratified tests, as described below.

**Table 2:** Associations of clinical/demographic variables with overall microbiota composition (β-diversity).

Variable	P-value^1^		
	All	Controls	Cases
Disease Status	0.00078	na	na
Enrollment age	5.00E-06	0.001213	0.003971
Ethnicity	0.18	0.2756	0.7073
Gender	0.57	0.4096	0.4352
Breastfeeding	0.00025	0.07256	0.03466
Mode of delivery	0.43	0.123	0.9031
Maternal diet restriction	0.021	0.5322	0.06151
PPI use	na	na	0.05907
Gestational age	0.11	0.12	0.87
Birth weight	0.18	0.33	0.92
Enrollment weight	0.00014	0.00072	0.11
Presumed MPA	0.50	0.15	0.97
Other med	0.62	0.94	0.57
Abx	0.61	0.40	0.72
Zantac	0.010	na	0.98
Cereal	0.0025	0.24	0.39
Prune	0.25	0.28	0.51

^1^ P-value ascertained by permutational ANOVA (PERMANOVA)

**Figure 1:**
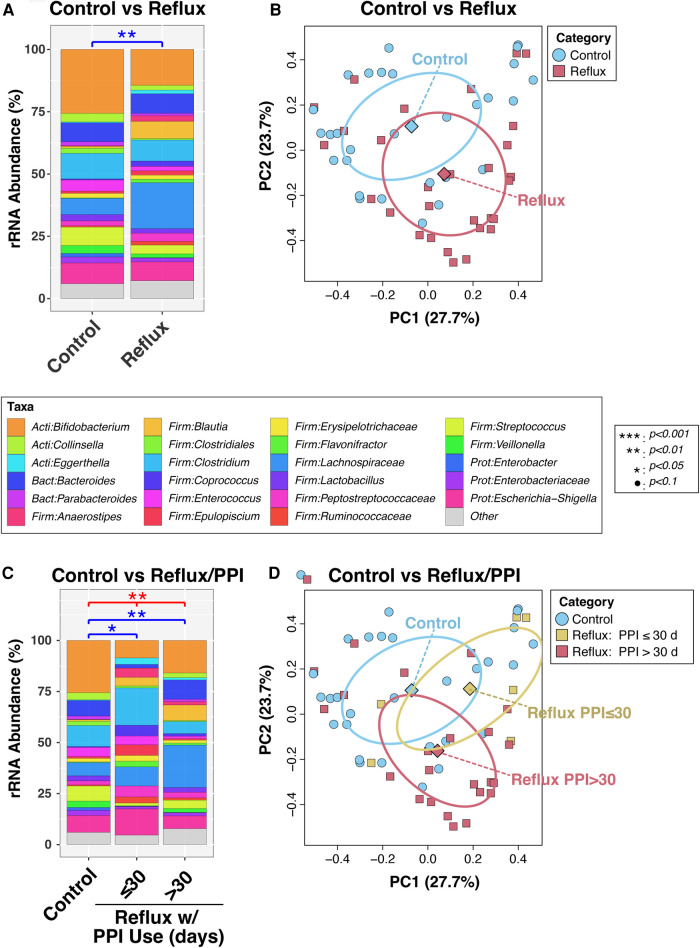
Intestinal microbiota differ in overall composition (β-diversity) with gastroesophageal reflux. Panels (**A**) and (**B**) compare subjects with and without reflux, while panels (**C**) and (**D**) stratify Reflux infants by PPI use. In panels (**A**) and (**C**), relative abundances of taxa are summarized at the genus level. Between-group differences in β-diversity were evaluated by permutational ANOVA, with significant results indicated by asterisks. The results of principal components analyses are shown in panels (**B**) and (**D**).

**Figure 2:**
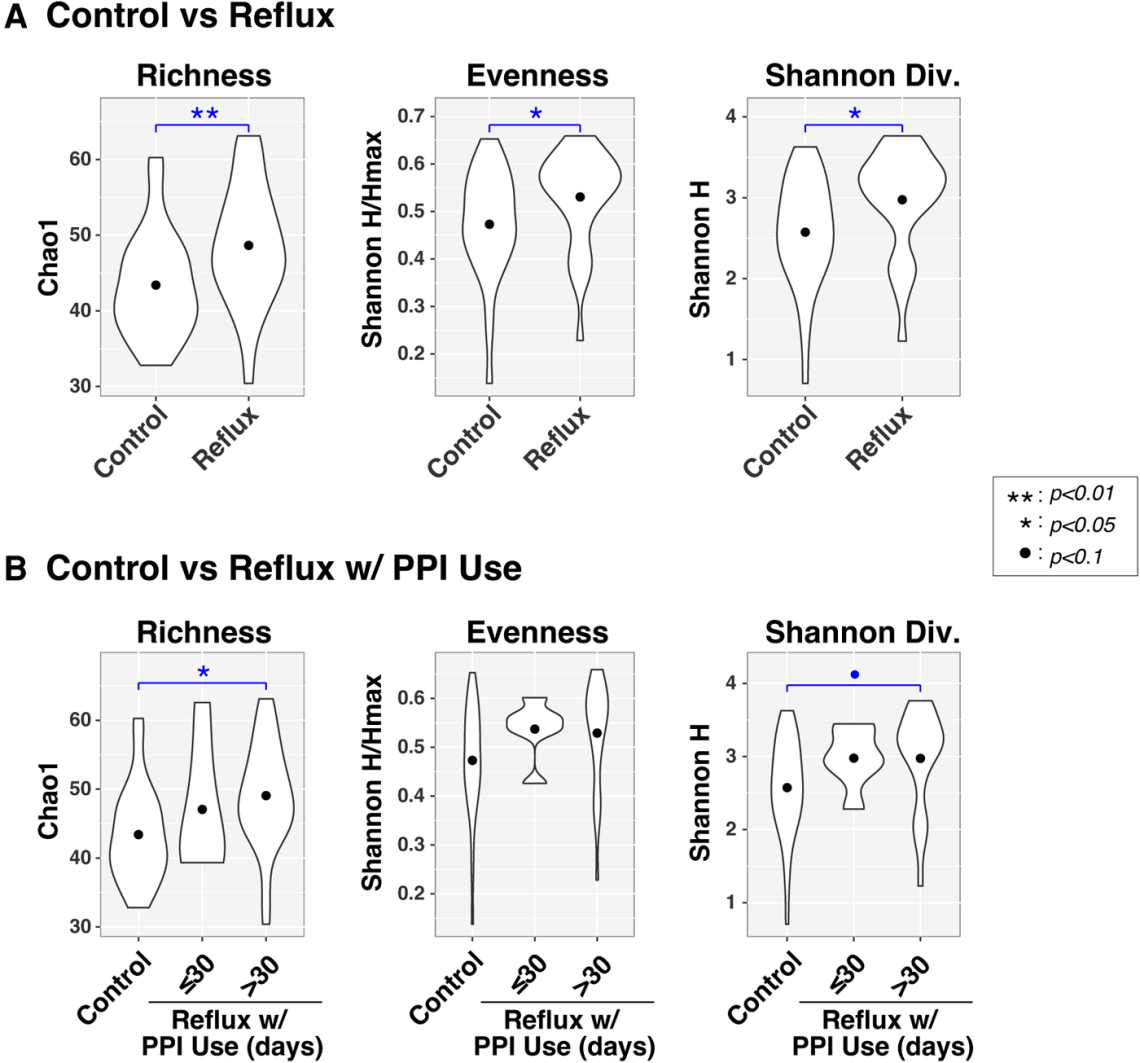
Microbial diversity (α-diversity) differs with gastroesophageal reflux. Violin plots compare richness (Chao1), evenness (Shannon H/Hmax), and Shannon diversity (H) of intestinal microbiota between infants with and without Reflux (panel **A**) and stratified by PPI use (panel **B**). Results of ANOVA tests are indicated by asterisks.

In a final bivariable PERMANOVA model, Reflux and Control infants differed significantly after adjusting for age (*P* = 0.0011; Figure 1A). This result was corroborated by PCoA analysis (Figure 2B), which demonstrated clustering of subjects by disease status, though some overlap was apparent between groups. Disease status remained significantly associated with microbiota (*P* = 0.012) when the PERMANOVA analysis was restricted to just the non-exclusively breastfed infants (*N* = 28 and 19 for Reflux and Control, respectively); only one Reflux infant was exclusively breastfed, so a similar stratified test could not be conducted among exclusively breastfed infants. Similarly, among infants who did not consume cereal (*N* = 9 and 27 for Reflux and Control, respectively), Reflux and Control infants differed significantly in β-diversity (*P* = 0.0096), whereas cereal consumption had no effect on the microbiota of Reflux infants (*P* = 0.89, comparing *N* = 9 vs. 20 Reflux infants not consuming vs. consuming cereal). Finally, only five Reflux infants did not receive H-2 antagonists; these infants did not differ significantly from either Reflux infants receiving H-2 antagonists (*P* = 0.98, *N* = 5 vs. 24) or Control infants (*P* = 0.15; *N* = 5 vs. 28), none of whom received H-2 antagonists.

We next analyzed the effects of PPI use among Reflux infants and in comparison, to Control infants. Reflux infants were dichotomized into those treated with PPI for ≤30 days (*N* = 6) vs. >30 days (*N* = 23). The microbiotas of these two groups differed significantly (*P* = 0.059) in a univariable PERMANOVA analysis, but not when age was added as a covariate (*P* = 0.60; Figure 1C). In contrast, both groups differed significantly from the Control group (*P* = 0.048 and 0.0029, for comparison of Control to Reflux infants receiving PPI for ≤30 days or >30 days, respectively) after adjusting for age (Figure 1C). These results are evident in a PCoA analysis (Figure 1D), in which the Control and Reflux w/PPI >30 days groups cluster apart, whereas Reflux w/PPI ≤30 days overlaps with both groups.

Like the β-diversity results, both disease status and PPI were associated with differences in α-diversity (Figure 2), as assessed by measures of richness (Sobs, counting observed taxa), evenness (Shannon H/Hmax, the uniformity of taxa distributions), and Shannon diversity (an overall measure of diversity combining both richness and evenness). All three α-diversity indices differed significantly (*P* < 0.05) between Control and Reflux infants, with the latter consistently demonstrating elevated diversity. These effects were more muted when Reflux infants were stratified by PPI use (≤30 days vs. >30 days), likely resulting from reduced power due to smaller groups sizes. The Control and Reflux w/PPI >30 days groups differed in richness (*P* = 0.023) and approached significance in Shannon diversity (*P* = 0.082). Although the Reflux w/PPI ≤30 days group did not differ significantly from Controls (or Reflux w/PPI >30 days), the mean values for all three α-diversity indices in this group were elevated relative to those of Controls.

**Figure 3:**
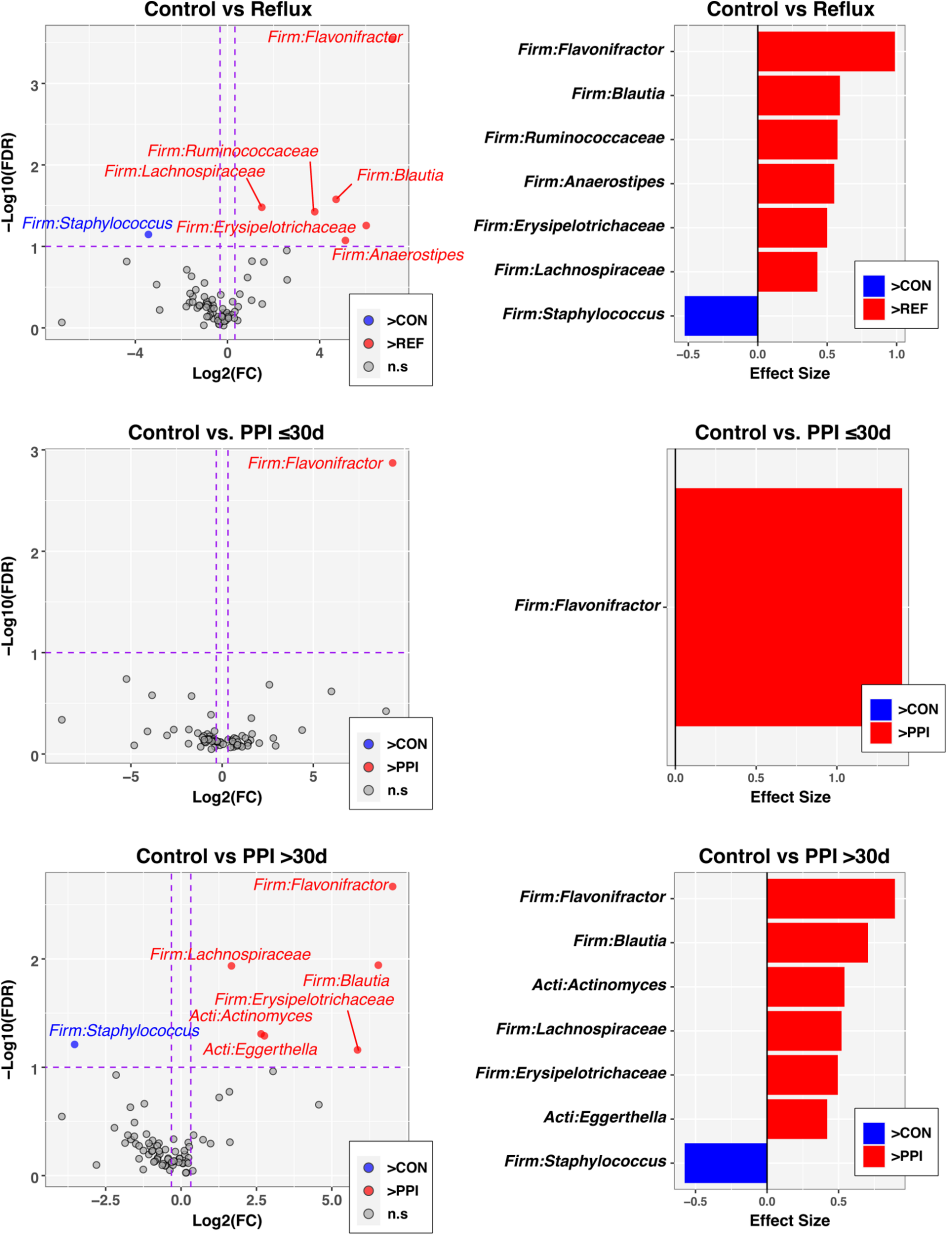
Individual taxa differing in relative abundance by Reflux status and PPI use. The left column of panels are volcano plots of fold-change (FC; Log2 transformed) vs FDR-corrected *p*-values (-Log10 transformed) ascertained by ALDEx2 analysis (See Methods). Vertical and horizontal dashed lines represent cutoffs of FC ≥1.5 and FDR-corrected *p*-value ≤0.1, respectively. The right column of panels are ALDEx2-calculated effect sizes of taxa meeting FC and FDR-corrected *p*-value cutoffs. In all panels, taxa enriched in the control group (CON) are highlighted in blue with FC and effect sizes less than zero, while taxa enriched in comparison groups (Reflux, PP ≤ 30 d, PPI > 30 d) are highlighted in red with FC and effect sizes greater than zero.

Finally, to identify individual taxa differing in relative abundance between groups defined by disease status and PPI use, we used the ANOVA-like differential expression (ALDEx2) R package (24), which considers the compositional nature of microbiota datasets. In these analyses, significance was defined by an FDR-corrected *p*-value ≤ 0.1 and absolute value of fold-change ≥1.25. Of the 76 genus-level taxa evaluated, seven met these criteria when comparing Control and Reflux groups (Figure 3, top panels), with six taxa having greater abundances in Reflux infants. The genus *Flavonifractor* also was significantly enriched in both the Reflux w/PPI ≤30 days and Reflux w/PPI >30 days groups compared to the Control group (Figure 3, middle and bottom panels). Furthermore, relative to Controls, PPI use >30 days was associated with elevated levels of the Actinobacteria *Actinomyces* and *Eggerthella*, in addition to most of the taxa identified in the Control vs. Reflux comparison. No taxa were significantly differentially abundant between PPI ≤30 days and PPI >30 days groups (data not shown).

## Discussion

The gut microbiome plays a vital role in the healthy state of the human host. While there is controversy about *in utero* influences, most studies report that mode of delivery, gestational age, and infant diet are the major driving forces for the developing infant microbiome (27, 28). Host genetics, environmental factors, and prior drug exposure also impact the development and maturation of gut microbiota in infants (5, 27). PPIs are a well described and efficacious therapy for GER treatment in adults. However, long-term use of PPIs has been associated with imbalances in gut microbiota as the PPIs may favor the colonization of specific microbial strains including *Enterococcus, Staphylococcus, Streptococcus, Micrococcus and Escherichia coli* (17, 18). Similar data on the effects of PPI exposure among infants is limited.

In this study, we performed a cross-sectional analysis and compared the composition and diversity of the fecal microbiome from 29 reflux infants treated with PPI therapy to that of 29 age-matched healthy control infants, the first such case-control comparison among infants up to 6 months of age. The complexity of the gut microbiota was compared between control and reflux infants stratified by age in months. As expected, younger untreated control infants harbored fewer microbiomes compared to older untreated control infants, as evidenced by lower α–diversity indices, suggesting a less developed microbiome. Conversely, there was significantly increased α–diversity among reflux infants overall, and particularly those less than or equal to 2 months of age, (Figure 2B). The higher proportion of breast-fed infants in the control group and higher proportion of formula fed infants in the reflux group (Table 1) is consistent with the significantly positive role of breastfeeding for the digestive health infants, as reported in other studies (29). Further, the prevalence of reflux in our study cohort is positively correlated with prematurity and lower birth weight consistent with previously published data (30). In addition, the majority of infants with reflux in this study were previously exposed to either solid food (cereal) or an H2-antagonist medication (Table 1).

The composition of gut microbiota among reflux infants substantially differed from that of control infants, particularly among reflux infants treated with PPI for 30 days or more (Figure 1). Microbiome composition in our study was significantly correlated with factors including disease status, age of the infant and maternal and infant dietary exposure. Among reflux subjects, we observed a significant correlation between PPI exposure and β-diversity in the older infants ages 3–6 months. In the study by Castellani et al, a longitudinal study of 12 infants on PPI therapy, the authors report a non-significant increase in diversity but ultimately conclude there were no significant changes in the fecal microbiome of infants exposed to PPIs (31). In our study, infants with reflux showed higher complexity and greater abundance of microbial taxa (Figure 2). In contrast a prior study noted that PPI exposure reduced gut microbial diversity and favored the colonization of *Streptococcaceae* when stool specimen of healthy twins was examined (32).

Our analysis reflects a cross sectional comparison of one time point; however, we observed higher relative abundances in several taxa among infants with reflux when compared to the control group. In our cohort, PPI exposure resulted in higher relative abundances of taxonomic groups including *Flavonifractor, Blautia, Ruminococcaceae, Anaerostipes, Erysipelotrichaceae*, *and* Lachnospiraceae among reflux infants with longer PPI exposure for more than 30 days (Figure 3). We noted increased proportions of *Flavonifractor* among reflux infants with shorter PPI exposure for less than 30 days. While long term exposure of PPIs in young infants may change the composition of gut microbiota, some of the changes may have a protective outcome. Lachnospiraceae microbes reportedly help in metabolizing sugars into short chain fatty acids although specific taxa of Lachnospiraceae, including *Blautia*, are increased in multiple inflammatory diseases including metabolic syndrome, obesity, diabetes, liver diseases, inflammatory bowel diseases, and chronic kidney disease (33, 34). *Flavonifractor plautii* and Lachnospiraceae *bacterium* are more abundant among children with irritable bowel syndrome (35). It is well described that gut microbiota naturally diversify as an infant grows (36, 37) and our findings suggest that PPI exposure may contribute to a more rapid diversification of the microbiome. Among control infants the composition was largely driven by an abundance of *Bifidobacterium* which was expected in this cohort that had more than a third of exclusively breastfed infants (38). Further only 7% of control infants had advance their diet to complementary feeding with cereal compared to 70% of reflux infants.

The findings in our study are significant. Gastroesophageal reflux is a highly prevalent condition among infants. Almost half of all newborn infants have regurgitation at least once a day and 40% of the infants under 4 months of age have frequent regurgitation and spitting up after feeding (39). Most infants do well with conservative measures including modified feeding volumes, thickened feeds, and position changes. Reflux symptoms gradually resolve and diminish by 12 months of age. However, some infants can have more significant symptoms of GERD and empirical use of PPI therapy is instituted when other measures are exhausted (40). Our data shows that infant with reflux treated with PPIs, have increased diversity and greater abundances of several bacteria taxa. The consequences of early gut microbiome maturation due to PPI exposure in infants are unknown. Although, improved diversity in gut microbiota is generally considered beneficial for overall health (41), early gut microbiota maturation in very young infants due to long-term exposure of PPIs may also contribute towards an imbalance or dysbiosis due to a change in the expected temporal microbiota colonization pattern. Similar to other exposures, PPI use may also cause a disturbance in immune system homeostasis and risk of allergic reactions in this age group (42, 43) Hence, further studies are recommended to elucidate the possible beneficial and/or detrimental effects of early gut microbiome maturation in young infants exposed to PPI therapy for reflux.

There are several limitations in this study. First, our findings are derived from fecal samples and microbial samples from other parts of the gastrointestinal tract (GIT) were not analyzed. Hence, the impact of PPIs on commensals living in the upper GIT and oral microbiome was not investigated. The smaller size of the sample in our study may affect outcomes of some correlations. Finally, we did not observe the impact of PPIs treatment cessation on gut microbiome composition in the reflux group and could not perform a longitudinal follow up study to investigate the long-term impact of early gut microbiome maturation in young infants.

In conclusion, we present the first case-control study comparing the gut microbiome diversity and abundance in young infants with and without prior exposure to PPIs. Like previous studies among adults, we report significant changes to α- and β-diversity among reflux infants compared to age-matched controls. The subsets of control and reflux infants in this cohort were not evenly matched for some clinical variables (described in Table 1). Infants in the control group were younger at enrollment and had a much higher proportion of breast-fed infants (35.8% vs. 3.5% among reflux infants). In addition, reflux infants had higher proportions of premature births, cesarean delivery and formula feeding. However, after adjusting for age and exclusive breastfeeding the two factors that significantly influenced the microbiome composition were PPI exposure by age in months and the duration of PPI use in days.

## Data Availability

The datasets presented in this study can be found in online repositories. The names of the repository/repositories and accession number(s) can be found below: https://www.ncbi.nlm.nih.gov/bioproject; PRJNA887191.
